# Effect of Referral Patterns and Treatment Type on Oncologic Outcomes for Women with Ductal Carcinoma In Situ

**DOI:** 10.7759/cureus.1128

**Published:** 2017-03-31

**Authors:** Elaine S Wai, Mary Lesperance, Linghong Lu, Cheryl S Alexander, Pauline T Truong

**Affiliations:** 1 Radiation Oncology, University of British Columbia, BC Cancer Agency; 2 Statistics, University of Victoria; 3 BC Cancer Agency

**Keywords:** ductal carcinoma in situ, breast cancer, mastectomy, radiation therapy, breast conservation, local recurrence, outcomes, prognostic factors, referral

## Abstract

**Objective:**

Management of ductal carcinoma in situ (DCIS) remains controversial. This study examined long-term outcomes in a population-based cohort of patients with pure DCIS treated with breast-conserving surgery (BCS) alone, BCS + radiotherapy (RT), and mastectomy. Outcomes were compared between patients referred versus not referred for oncologic assessment after definitive surgery.

**Materials and methods:**

Subjects were 2575 women diagnosed between 1985 and 1999. Data from several electronic databases were linked and analyzed. Outcomes were invasive local recurrence-free survival (ILRFS), mastectomy-free survival (MFS), breast cancer-specific survival (BCSS), and overall survival (OS).

**Results:**

Median follow-up time was 9.8 years. Overall, 56% (n = 1448) of subjects were referred to a cancer centre. Factors associated with non-referral were older age, comorbidities, and travel distance. Ten-year MFS, BCSS, and OS were higher among referred patients (all p ≤ 0.001). In cohorts treated with BCS alone (n = 1314) vs. BCS + RT (n = 510) vs. mastectomy (n = 751), 10-year ILRFS were 93.7% vs. 96.6% vs. 97.7%, (p < 0.001) and BCSS were 97.6% vs. 99.8% vs. 98.6%, (p = 0.01). Corresponding rates of ipsilateral invasive breast relapse at 10 years were 6.3% after BCS alone, 3.4% after BCS + RT, and 2.3% after mastectomy (p < 0.001). On multivariable analysis, factors associated with improved ILRFS were older age at diagnosis, low comorbidity score, absence of comedo histology, mastectomy, and post-BCS RT.

**Conclusion:**

Patients with DCIS referred for oncologic assessment were more likely to undergo post-BCS RT, resulting in lower mastectomy and higher survival rates compared to non-referred patients. Patients with significant comorbidities were less likely to be referred and experienced lower ILRFS and BCSS. Referral for multidisciplinary oncologic assessment after surgery is warranted to individualize management and optimize outcomes for patients with DCIS.

## Introduction

Ductal carcinoma in situ (DCIS) comprises approximately 20-30% of mammographically detected breast cancers [1]. Management of DCIS has been the subject of randomized trials, prospective series, and retrospective reviews [2-12]. Diverse treatment strategies exist, including breast conserving surgery (BCS) alone, BCS plus radiotherapy (RT), or mastectomy, showing similar breast cancer-specific survival [2-12]. While there are no randomized trials directly comparing mastectomy to breast-conserving therapy in women with pure DCIS, the management of DCIS has evolved over time towards the increased use of breast conserving therapy for the majority of patients diagnosed in contemporary practice [10].

Among women who undergo BCS, randomized trials have shown that RT significantly reduces invasive and in-situ recurrence [2-3]. Prospective studies evaluating outcomes in patients treated with BCS alone have reported varied results [4-5]. Guidelines from the National Health Institute and American College of Radiology support consideration of BCS + RT or mastectomy as effective local therapies for most patients with DCIS and advocate continued research to evaluate the question of whether there are subsets with sufficiently low risk who may be treated with BCS alone [8-9].

While DCIS is a highly curable disease, invasive local recurrences can compromise survival [13-15]. Despite randomized trials demonstrating local control benefits with RT after BCS, the use of RT varies widely at the population level [15]. In addition, even though evidence-based guidelines advocate multidisciplinary evaluation for patients with DCIS [7-9]  and quality indicators have been developed to monitor population-based treatment [14], disparities in care delivery among different populations are still documented [15]. These findings raise questions regarding whether variations among community physicians in referring patients for oncologic assessment after diagnosis may impact treatment and outcomes for women with DCIS.

The British Columbia Cancer Agency (BCCA) is a provincial organization that operates six regional cancer centres (four during the study era), to serve a population of approximately 4 million people. It provides 100% of the RT and manages the budget for all antineoplastic drugs in British Columbia (BC). Women diagnosed with breast cancer are typically seen by surgeons for definitive surgery, then referred to one of the regional cancer centres if felt necessary by the surgeon or family physician for a discussion of adjuvant therapy. Patients unsure of which initial surgery to pursue or patients with challenging management decisions may also be referred for review prior to surgery.

The primary objectives of the current study were to compare differences in baseline characteristics and outcomes among women with DCIS according to referral status and treatment. The secondary objectives were to identify factors associated with ipsilateral invasive local recurrence and breast cancer-specific survival.

## Materials and methods

### Study subjects

A retrospective study was conducted of 2575 women with newly diagnosed pure DCIS between January 1, 1985 and December 31, 1999, treated in the province of British Columbia, Canada. Exclusion criteria were concomitant or previous invasive cancer of any site, or contralateral DCIS predating the index diagnosis.

### Data sources

Cases were identified using the BC Cancer Registry. These data were linked to electronic files from the Screening Mammography Program of BC (SMPBC), the Hospital Separation File/Discharge Abstract Database, Vital Statistics Agency, Ministry of Health, Government of British Columbia, and the BCCA Breast Cancer Outcomes Unit. The SMPBC database, which contains demographic, prognostic, and initial treatment information on all women screened since 1988, maintains active follow-up to ascertain the final pathology results to identify all screen-detected and interval cancers. The Hospital Separation File/Discharge Abstract Database File contains data from discharge summaries of all hospitalizations and day surgeries in BC. The BC Ministry of Health and Vital Statistics owns and has approved access and use of the data facilitated by Population Data BC for this study. For patients diagnosed between 1985 and 1988, there was no SMPBC data to supplement the other data sources.

### Study setting and institutional guidelines

The BCCA database prospectively collects demographic, clinicopathologic, treatment, and outcomes data for all women with newly diagnosed in situ or invasive breast cancer referred for management. Central pathology review was available by request of the treating oncologist. If not requested, pathology was reported by pathologists in the community or at academic centres in Vancouver or Victoria.

Institutional management guidelines for DCIS were available and regularly updated by the interdisciplinary Breast Tumour Group, comprised of all surgeons, radiation oncologists, and medical oncologists treating breast cancer at the BCCA. In the early years of the study, a high proportion of patients with DCIS underwent mastectomy. BCS was advised if the DCIS was unifocal, without comedo histology, and the patient was suitable for follow-up (clinical exam and mammogram “easy to interpret”). In 1993, following the publication of the NSABP B17 study, provincial guidelines were updated to recommend adjuvant breast RT after BCS for patients with DCIS  >1 cm, comedocarcinoma, or margins <5 mm. Women with well-differentiated DCIS <1 cm and clear margins >5 mm were generally managed by wide excision alone. Women with diffuse DCIS (>5 cm or > ¼ of the breast on mammogram) were recommended to undergo mastectomy. Tamoxifen was not recommended during the study era outside of available clinical trials. Provincial follow-up guidelines recommended history and physical exam by a physician every three-six months for the first five years, then annually, with annual mammography.

### Outcomes analysis

Primary outcomes were ipsilateral invasive local recurrence-free survival (ILRFS) and breast cancer-specific survival (BCSS). Secondary outcomes were mastectomy-free survival (MFS), and overall survival (OS). Chi-square tests were used to compare clinicopathologic and treatment characteristics among cohorts treated with BCS alone (n = 1314), BCS + RT (n = 510), and mastectomy (n = 751); and among cohorts who were referred (n = 1448) versus not referred (n = 1127) for oncologic assessment after definitive surgery. Kaplan-Meier (KM) estimates and log-rank tests were used to compare survival outcomes by treatment type and by referral status. Multivariable Cox regression analysis was performed to assess predictors of ILRFS and BCSS. Factors included in the multivariable model included age, referral to BCCA status, Deyo-Charlson Comorbidity score [16], nuclear grade, size of primary tumour, margin status, comedo histology, and initial treatment. Estrogen receptor (ER) status was not included in the Cox modeling because testing for ER was not routine during the study era. Necrosis was not included because comedonecrosis was included independently in the histology code, and a large number of cases had unknown values for necrosis.

The study was approved by the BCCA Research Ethics Board.

## Results

Median follow-up time was 9.8 years. Overall, 10-year ipsilateral invasive breast relapse was 4.5% and BCSS was 98.3%.

### Comparisons of clinical characteristics and outcomes by referral status

Overall, 1448 (56%) women were referred to BCCA after primary surgery. Referral rates consistently approximated 50% from 1985 until 1996, after which referral rates gradually increased from 57% in 1996 to 69% in 1999. Referred patients were more likely to be younger at diagnosis, live within two hours from the nearest cancer centre at the time of diagnosis, and have lower comorbidity scores (Table 1).

Multivariable analysis showed referral status was associated with age, Deyo-Charlson comorbidity scores, and residence > 2 hours from the nearest cancer centre at the time of diagnosis. Those less likely to be referred had a higher incidence of unknown grade, lesion size, and margin status. Those with comedocarcinoma were more likely to be referred.

Among women who were not referred, the majority (75%) underwent BCS alone while 24% underwent mastectomy. Among women who were referred, treatment type was evenly divided among the three groups (Table 1).

**Table 1:** Clinicopathologic characteristics of the entire cohort and comparisons by referral status and treatment type. BCCA: British Columbia Cancer Agency; BCS: Breast conserving surgery; RT: Radiation therapy.

		All patients (n = 2575)	Referred to BCCA (n = 1448)	Not referred (n = 1127)	p-value	BCS alone (n = 1314)	BCS + RT (n = 510)	Mastectomy (n = 751)	p-value
		n (%)	n (%)	n (%)		n (%)	n (%)	n (%)	
Age (years)									
	<50	757 (29)	484 (33)	273 (24)	<0.001	346 (26)	161 (32)	250 (33)	<0.001
	50-59	657 (26)	396 (27)	261 (23)		321 (24)	170 (33)	166 (22)	
	60-69	620 (24)	336 (23)	284 (25)		312 (24)	111 (22)	197 (26)	
	≥70	541 (21)	232 (16)	309 (27)		335 (26)	68 (13)	138 (18)	
	Median	57	55	61		59	55	57	
Referral status	Referred					461 (35)	510 (100)	477 (64)	<0.001
	Not referred					853 (65)	0	274 (37)	
Travel time to BCCA centre	≤2 hours	1787 (69)	1205 (83)	582 (52)	<0.001	817 (62)	422 (83)	548 (73)	<0.001
	>2 hours	501 (20)	243 (17)	258 (23)		285 (22)	88 (17)	128 (17)	
	Unknown	287 (11)	0	287 (26)		212 (16)	0	75 (10)	
Comorbidity score	0	1021 (40)	625 (43)	396 (35)	<0.001	525 (40)	254 (50)	242 (32)	<0.001
	1	152 (6)	94 (7)	58 (5)		78 (6)	37 (7)	37 (5)	
	2	836 (33)	474 (33)	362 (32)		392 (30)	161 (32)	283 (38)	
	≥3	566 (22)	255 (18)	311 (28)		319 (24)	58 (11)	189 (25)	
Nuclear grade	1	582 (23)	331 (23)	251 (22)	<0.001	394 (30)	84 (17)	104 (14)	<0.001
	2	767 (30)	528 (37)	239 (21)		357 (27)	197 (39)	213 (28)	
	3	636 (25)	441 (31)	195 (17)		180 (14)	195 (38)	261 (35)	
	Unknown	590 (23)	148 (10)	442 (39)		383 (29)	35 (7)	173 (23)	
Size of primary tumor	≤1.5 cm	1236 (48)	737 (51)	499 (44)	<0.001	758 (58)	270 (53)	208 (28)	<0.001
	1.6 - 4 cm	634 (25)	452 (31)	182 (16)		210 (16)	193 (38)	231 (31)	
	> 4 cm	309 (12)	189 (13)	120 (11)		101 (8)	32 (6)	176 (23)	
	Unknown	396 (15)	70 (5)	326 (29)		245 (19)	15 (3)	136 (18)	
Margin status	Negative ≥2 mm	1785 (69)	1150 (79)	635 (56)	<0.001	827 (63)	390 (77)	568 (76)	<0.001
	Close <2 mm	271 (11)	99 (7)	172 (15)		176 (13)	45 (9)	50 (7)	
	Positive	268 (10)	125 (9)	143 (13)		151 (12)	66 (13)	51 (7)	
	Unknown	251 (10)	74 (5)	177 (16)		160 (12)	9 (2)	82 (11)	
Comedo features	Absent	1590 (62)	837 (58)	753 (67)	<0.001	956 (73)	252 (49)	382 (51)	<0.001
	Present	985 (38)	611 (42)	374 (33)		358 (27)	258 (51)	369 (49)	
Initial treatment	BCS	1314 (51)	461 (32)	853 (76)	<0.001				
	BCS + RT	510 (20)	510 (35)	0					
	Mastectomy	751 (29)	477 (33)	274 (24)					

**Figure 1:**
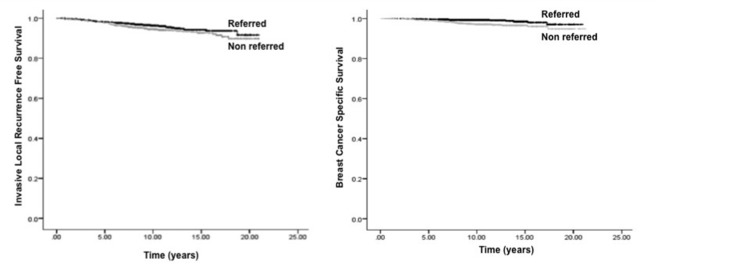
Comparisons of Kaplan-Meier. (A) Invasive local recurrence-free survival and (B) breast cancer-specific survival by referral status.

Compared to non-referred patients, referred patients had a non-significant trend for higher 10-year ILRFS (96.3% vs. 94.4%, p = 0.07) (Figure 1A) and significantly higher 10-year BCSS (99.3% vs. 97.1%, p = 0.001) (Figure 1B), MFS (56.9% vs. 51.7%, p < 0.001), and OS (91.4% vs. 85.8%, p < 0.001).

### Comparisons of clinical characteristics and outcomes by treatment type

During the study era, mastectomy use gradually decreased and post-BCS RT use increased. Women aged >50 years and women with increased comorbidities had higher rates of mastectomy, while women aged >70 years and women who lived more than two hours drive from a cancer centre were more likely to be treated with BCS alone (Table 1).

**Figure 2:**
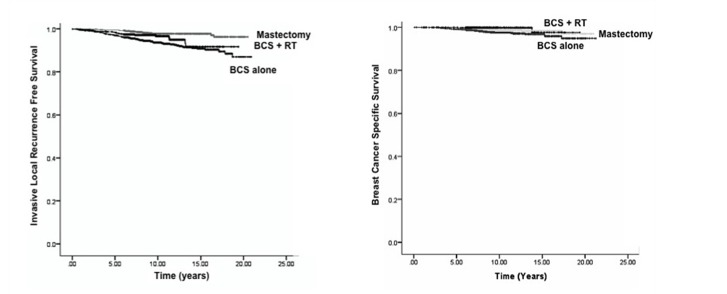
Comparisons of Kaplan-Meier. (A) Invasive local recurrence-free survival and (B) breast cancer-specific survival by treatment type.

In cohorts treated with BCS alone vs. BCS + RT vs. mastectomy, 10-year outcomes were: ILRFS 93.7% vs. 96.6% vs. 97.7%, (p < 0.001), (Figure 2A); BCSS 97.6% vs. 99.8% vs. 98.6%, (p = 0.01), (Figure 2B); and OS 86.5% vs. 93.5% vs. 90.1%, (p < 0.001). Ten-year MFS rates were lower in women treated with BCS alone compared to BCS + RT (72.2% vs. 87.2%), (p < 0.001).

Ten-year invasive local recurrence rates, stratified by clinicopathologic characteristics and treatment type are shown in Table 2.

**Table 2:** Stratified analysis of 10-year invasive local recurrence rates (rate% (standard deviation)) by treatment type. BCS: Breast conserving surgery; RT: Radiation therapy.

		BCS alone	BCS + RT	Mastectomy	Log-rank p-value
Age (years)	<50	5 (0.013)	6 (0.024)	4 (0.013)	0.371
	50-59	9 (0.018)	1 (0.008)	0 (0.000)	<0.001
	60-69	6 (0.015)	0 (0.000)	1 (0.007)	<0.001
	≥70	5 (0.013)	3 (0.021)	4 (0.018)	0.428
Referral status	Not referred	6 (0.009)	NA	2 (0.010)	0.005
	Referred	6 (0.012)	3 (0.009)	2 (0.007)	<0.001
Comorbidity score	0	0.4 (0.003)	0 (0.000)	0.4 (0.004)	0.607
	1	1 (0.013)	0 (0.000)	0 (0.000)	0.622
	2	10 (0.017)	6 (0.021)	2 (0.009)	<0.001
	≥3	12 (0.020)	7 (0.036)	5 (0.017)	0.003
Nuclear grade	Grade 1	5 (0.013)	3 (0.033)	3 (0.018)	0.441
	Grade 2	7 (0.014)	3 (0.012)	3 (0.012)	0.024
	Grade 3	7 (0.021)	2 (0.009)	2 (0.011)	0.002
	Unknown	7 (0.013)	9 (0.049)	2 (0.010)	0.002
Size of primary tumor	≤1.5 cm	7 (0.010)	2 (0.012)	3 (0.011)	<0.001
	1.6 - 4 cm	8 (0.020)	3 (0.013)	2 (0.011)	0.003
	>4 cm	4 (0.020)	7 (0.047)	3 (0.015)	0.583
	Unknown	5 (0.015)	7 (0.064)	0.8 (0.008)	0.004
Margin status	Negative	7 (0.010)	2 (0.011)	2 (0.006)	<0.001
	Positive/close/unknown	6 (0.011)	6 (0.023)	3 (0.013)	0.067
Comedocarcinoma	Absent/unknown	6 (0.008)	2 (0.016)	3 (0.009)	0.003
	Present	8 (0.015)	4 (0.012)	2 (0.008)	<0.001

### Multivariable analysis

On Cox regression analysis, factors associated with improved ILRFS were older age at diagnosis, low comorbidity score, absence of comedo histology, mastectomy, and post-BCS RT. Factors associated with improved BCSS were low comorbidity score, small tumor size, mastectomy, and post-BCS RT.

## Discussion

The question of whether referral for oncologic assessment and treatment allocation affect outcome is the focus of the current report. Our analysis found that initial treatment type affected local recurrence, subsequent mastectomy rates, and survival outcomes for women with pure DCIS. In British Columbia, however, women who would like to undergo breast conserving therapy including RT need to be referred to one of the BCCA centres for assessment and treatment. While referral to a BCCA centre was not associated with higher ILRFS or BCSS, those women who were not referred experienced higher mastectomy rates and lower rates of adjuvant RT.

Women were more likely to be referred to one of the cancer centres if they were younger, have fewer comorbid conditions, and lived closer to a cancer centre. Similarly, in a study of 4139 women with DCIS diagnosed between 1998 and 2005, Krotneva, et al. reported that age and distance from a cancer centre influenced the probability of referral [17]. Our study showed that women referred after surgery were more likely to receive RT after BCS and experienced higher MFS, BCSS, and OS compared to non-referred women, despite having higher risk pathologic features. The improved outcomes may be attributed to not only better baseline health status, but may also be related to more complete assessment and treatment, as shown by the higher rates of clear margins and post-BCS RT use amongst those referred to a cancer centre.

In the current study, the rate of invasive local recurrence of two percent after mastectomy was similar to outcomes reported in other studies [18]. The rates of ILR after BCS alone or BCS + RT of <10% were lower compared to data from randomized trials {6}, but similar to results from the lower risk arm of the recently published ECOG-ACRIN E5194 study of surgical excision without radiotherapy for low-risk breast patients [4]. The reasons for this observation are likely related to significant patient and treatment selection. Consistent with the literature, the rate of invasive recurrence was decreased by half with adjuvant radiotherapy in our study.

One concerning finding was the significantly lower ILRFS, BCSS, and OS among women with higher comorbidity scores, which persisted in multivariable modeling. Other studies have similarly observed that women with in situ and invasive breast cancer with comorbidities are treated less aggressively and have lower overall survival rates [19-21]. Some have also reported higher breast cancer mortality, even after controlling for other prognostic factors, such as stage and treatment [19-21]. In our study cohort, women with comorbidities were more frequently treated with mastectomy, and after controlling for differences in treatment, were still at higher risk for invasive local recurrence and breast cancer mortality. It appears that even in the setting of DCIS, patients with comorbidities should be assessed regarding definitive local therapy as other host or tumor biologic factors may be contributing to the observed poorer oncologic outcomes.

In the current multivariable analysis, factors associated with reduced risk of invasive local recurrence were older age at diagnosis, low comorbidity score, absence of comedonecrosis, mastectomy, and post-BCS RT. While these findings are consistent with other studies [22-23], these clinical factors were still not able to definitively discern those at very high or low risk of invasive recurrence. The Memorial Sloan Kettering Cancer Center developed a predictive model that estimates recurrence risk using factors such as age, use of RT, margin status, and a number of excisions [24], but independent evaluation of this model found suboptimal performance [25]. As reproducible models using purely clinical factors for DCIS risk prediction remain lacking, other methods such as the Oncotype DX DCIS score may prove useful in aiding clinicians to identify which patients will most benefit from which treatments [26-27].

The current study’s findings should be interpreted in the context of its limitations and strengths. As the analysis is retrospective, there were inherent biases in patient and treatment selection. While central pathology review was not performed in the entire population, a proportion of patients would have had pathology review, and the results from this study and previous studies of these data found that prognostic factors relevant in the literature were also relevant in this dataset [22-23, 28]. The local recurrence outcome examined only included invasive recurrences, as all invasive recurrence would have been captured, but in situ recurrences were not recorded for all patients. In addition, our dataset lacked estrogen receptor status which impacts adjuvant hormone therapy use. Despite these limitations, the study contributes outcomes data in a large population-based cohort of women treated in a universal access health care system with long-term follow-up. While treatment decisions were made by the clinicians and patients, provincial guidelines updated over time and interdisciplinary tumor board conferences were available to guide decisions.

## Conclusions

In this large population cohort, patients with DCIS referred for oncologic assessment were more likely to undergo post-BCS RT, resulting in lower rates of mastectomy and higher rates of survival compared to non-referred patients. Patients with comorbidities were less likely to be referred and were more likely to experience invasive recurrence and poorer BCSS. As DCIS is a complex but eminently curable disease, referral for multidisciplinary oncologic assessment after definitive surgery is warranted to discuss and individualize management for women with DCIS.
